# Lipid Profile in Patients With Primary Ovarian Insufficiency: A Systematic Review and Meta-Analysis

**DOI:** 10.3389/fendo.2022.876775

**Published:** 2022-06-10

**Authors:** Ling Huang, Hanfeng Wang, Minglu Shi, Weizheng Kong, Mei Jiang

**Affiliations:** ^1^ School of Chinese Medicine, Beijing University of Chinese Medicine, Beijing, China; ^2^ School of Acupuncture-Moxibustion and Tuina, Beijing University of Chinese Medicine, Beijing, China; ^3^ Beijing Research Institute of Chinese Medicine, Beijing University of Chinese Medicine, Beijing, China

**Keywords:** primary ovarian insufficiency, lipid profile, cardiovascular disease, meta-analysis, dyslipidemia

## Abstract

**Backgrounds:**

A large number of studies have investigated the effect of early menopause on cardiovascular disease (CVD) outcomes and the relationship between the levels of lipid profile and primary ovarian insufficiency (POI). However, the results are inconsistent. The aim of this meta-analysis was to assess whether the levels of total cholesterol (TC), triglyceride (TG), high density lipoprotein (HDL) and low density lipoprotein (LDL) changed in women with POI relative to healthy controls.

**Methods:**

To identify eligible studies, references published prior to December 2021 were searched in the PubMed, Embase, Cochrane Library and Web of Science databases. DerSimonian-Laird random-effects model was used to estimate the overall standard mean difference (SMD) between POI and healthy control subjects. Subgroup analysis and sensitivity analysis were preformed, and publication bias was assessed.

**Results:**

A total of 12 studies featuring 846 women with primary ovarian insufficiency and 959 healthy women were selected for analysis. The meta-analysis showed that the levels of TC (SMD: 0.60; 95% CI: 0.32 to 0.89; *P*<0.0001), TG (SMD: 0.36; 95% CI: 0.12 to 0.60; *P=*0.003), LDL (SMD: 0.46; 95% CI: 0.16 to 0.76; *P=*0.003) were significantly increased in women with POI. There was no significant change in the level of HDL (SMD: 0.25; 95% CI: -0.12 to 0.61; *P=*0.19). Subgroup analysis showed that the heterogeneity in this meta-analysis of the correlation between lipid profile and POI might come from by region, sample size, number of cases, mean body mass index (BMI) value of cases and mean age of cases.

**Conclusions:**

Scientific evidence suggests that the lipid profile levels were altered in patients with primary ovarian insufficiency compared to healthy controls. Therefore, we recommend that early medical intervention (e.g., hormone replacement therapy) to minimize the risk of CVD morbidity and mortality associated with dyslipidemia in patients with POI.

**Systematic Review Registration:**

PROSPERO, identifier CRD42021297088

## Introduction

Cardiovascular disease (CVD) is the leading cause of mortality among women worldwide (1). The incidence of CVD increases significantly after menopause but is rarely seen in premenopausal women (2). Postmenopausal women are at increased risk of cardiovascular and metabolic diseases because of the deficiency of estradiol and the central distribution of body fat (3).

The mechanism of postmenopausal dyslipidemia leading to CVD has been clarified (1). Studies have confirmed that the levels of serum total cholesterol (TC), triglyceride (TG) and low density lipoprotein (LDL) increased in postmenopausal women, while the level of serum high density lipoprotein (HDL) decreased (4). Elevated level of serum LDL is often regarded as a sign of hyperlipidemia, which increases the risk of coronary heart disease. Moreover, the incidence of atherosclerosis increases with the level of LDL as a result of oxidative modification of LDL. Oxidized LDL deposits on the arterial wall of cardio-cerebral vascular areas and gradually forms atherosclerotic plaques. The atherosclerotic plaques block the corresponding blood vessels, lead to atherosclerosis and increase the risk of CVD (5). Studies have shown that even if the level of serum LDL is normal, partial oxidation which leads to atherosclerosis cannot be ruled out (6). In addition, elevated levels of serum TC and TG are also the factors of the deficiency in antioxidant mechanism, which contributes to subclinical atherosclerosis (7). Therefore, these changes are partly responsible for the increased incidence of CVD in postmenopausal women.

A number of studies have investigated the effect of earlier age at menopause on CVD outcomes (8–10). Compared to women with menopause at the age of 49~55 years, women with early menopause have an approximately 80% increased risk of mortality from CVD (11). Primary ovarian insufficiency (POI), which is a typical disease characterized by early menopause (before age 40years) in women, is defined as amenorrhea for four months and an elevated follicle stimulating hormone (FSH) level>25IU/L (measured twice at least four weeks apart) in women under 40 years old (12). Hence, POI, by virtue of early menopause and chronic estradiol deficiency, is a risk factor for CVD.

Knowledge of the potential associations of lipid profile with diminished ovarian response to FSH has public health significance for reducing the risk of cardiovascular morbidity and mortality in women. POI may provide important information to disentangle the effects of menopause and age on lipid profile. Thus, this systematic review and meta-analysis aimed to evaluate the levels of serum lipid profile in women with POI and healthy controls.

## Materials and Methods

### Reporting Guidelines

This systematic review and meta-analysis was designed according to the Preferred Reporting Items for Systematic Reviews and Meta-Analyses (PRISMA) statement (13), and was prospectively registered on PROSPERO (registration number: CRD42021297088). This study was conducted according to the Meta-analysis of Observational Studies in Epidemiology (MOOSE) guidelines (14).

### Search Strategy

The PICO (population/intervention/comparison/outcome) components were as follows: P (women before the age of 40), I (women with primary ovarian insufficiency), C (healthy women), O (serum levels of lipid profile including TC, TG, HDL, and LDL). To identify eligible studies, an exhaustive literature search was performed in the PubMed, Embase, Cochrane Library and Web of Science databases (with language was restricted to English; without restricting by location and journal) through December 2021 to identify published studies using the following keywords: “primary ovarian insufficiency” OR “premature ovarian insufficiency” OR “premature ovarian failure” OR “premature menopause” OR “POI” OR “POF” OR “menopause premature” OR “early menopause” AND “lipid profile” OR “total cholesterol” OR “TC” OR “triglycerides” OR “TG” OR “high-density lipoprotein” OR “HDL” OR “low-density lipoprotein” OR “LDL”.

### Inclusion and Exclusion Criteria

The inclusion criteria for eligible studies were as follows: (1) original human observational studies (case-control, cross-sectional, or longitudinal design); (2) all subjects involved in studies had no gynecological surgery or undergone therapy that could induce menopause; (3) studies focusing on the association between at least one of the serum lipid profile levels including TC, TG, HDL, and LDL with POI; and (4) studies included data on the levels of serum lipid profile for patients with POI and healthy individuals.

The exclusion criteria were as follows: (1) laboratory or animals studies; (2) reviews or case reports; (3) repeated publications; (4) studies without healthy control groups; (5) studies not providing exact data on the levels of serum lipid profile; (6) studies involved subjects were older than 40 years; and (7) studies with sample size <10.

### Study Selection and Data Extraction

**Figure 1:**
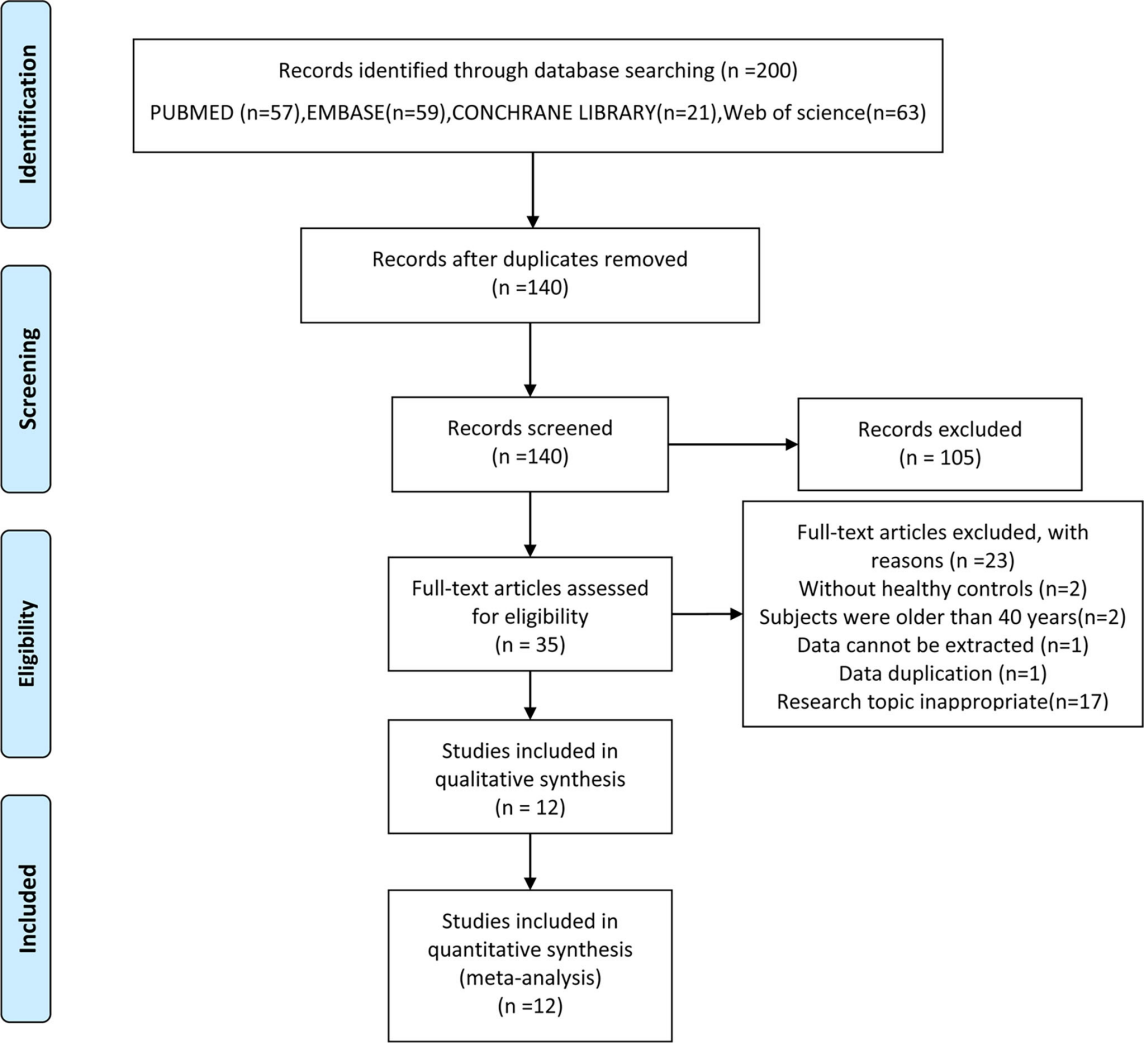
Flow chart of the selection process. From (15).

Literature screening and data extraction were independently done by two researchers (LH and HW). Differences between two reviewers were addressed in consultation with a third reviewer (MJ). Firstly, references were imported into EndNote, and duplicates were identified. Secondly, literature titles and abstracts were screened, and those did not meet the inclusion criteria were excluded. Finally, the full text of the references were read, and the studies were further excluded according to the inclusion and exclusion criteria. PRISMA flowchart explaining the selection process was shown in **Figure 1**. The data including the levels of serum TC, TG, HDL and LDL [mean ± standard deviation (SD)] were extracted from the references, and all data were rechecked by MJ.

### Quality Assessment

The quality assessment of eligible studies was performed according to the Newcastle-Ottawa Scale (NOS) star system (16), and all included studies were rated above 7.

### Statistical Analysis

The extracted data from the included studies were analyzed with a meta-analysis. Since the existing references were not consistent with regard to units, the pooled standardized mean difference (SMD) and 95% confidence intervals (CI) were used to determine the associations between lipid profile and POI. Heterogeneity in the studies was tested using Cochran’s Q two-sided test of homogeneity (17). If I-square (*I^2^*) <50%, a Mantel-Haenszel fixed-effect model was used, otherwise, a DerSimonian-Laird random-effects modelwas used (18, 19). Subgroup analysis was performed to identify associations between the levels of serum lipid profile and the characteristics of studies to examine if this could explain heterogeneity.Sensitivity analysis was performed to test the robustness of the pooled SMD by excluding each study. Funnel plot method (in cases where the number of included references were ≥ 10) was used to test publication bias. All analyses were performed using RevMan software, and *P*<0.05was considered to be statistically significant.

## Results

### Literature Search

From the initial 140 articles, 105 were excluded after reading titles and abstracts because they did not meet the inclusion criteria. 35 articles were included for full-text assessment, from which 23 were excluded: two without healthy controls; two involved subjects were older than 40 years; one involved data cannot be extracted; one repeated publication; seventeen research topic inappropriate. A total of 12 eligible papers were included (**Figure 1**), all of the studies featured a case-control design and included individual data from 846 cases and 959 healthy controls (20–31). The baseline characteristics, such as author, year, region, number of case/control subjects, age, body mass index (BMI), lipid profile and primary conclusion included in the studies were shown in **Table 1**.

**Table 1:** Summary characteristics of studies and participants.

No.	Study	Region	Women (n) (Cases vs.Control)		Age (years) (Cases vs. Control)		Age at diagnosis of POI (years)	BMI (kg/m^2^) (Cases vs. Control)		HRT/OC used		Lipid profile	Primary conclusion
			POI	Control	POI	Control		POI	Control	POI	Control		
1.	Falsetti (1999) (25)	Italy (Brescia)	40	30	32.6 ± 7.3	35 ± 3.5	NA	22.9 ± 3.8	22.2 ± 2.2	NA	NA	TC,LDL,HDL	Serum TC and LDL increased, serum HDL decreased in POI
2.	Ostberg (2005) (30)	Britain (London)	31	30	33.0 ± 7.0	33.5 ± 6.5	NA	22.9 ± 3.4	23.7 ± 3.2	No	No	TC,TG,LDL,HDL	Serum TG increased, serum TC, LDL and HDL remained unchanged in POI
3.	Knauff (2008) (28)	Netherlands (Utrecht,Rotterdam)	90	198	33.8 ± 5.6	30.3 ± 2.9	31.8 ± 6.3	24.9 ± 4.3	24.3 ± 4.8	No	No	TC,TG,LDL,HDL	Serum TG increased,serum HDL decreased, serum TC and LDL remained unchanged in POI
4.	Gulhan (2012) (26)	Turkey (Izmir)	47	60	36.8 ± 1.8	36.0 ± 2.3	NA	25.9 ± 4.9	24.8 ± 4.6	No	No	TC,TG,HDL,LDL	Serum TC and LDL increased, serum TG and HDL remained unchanged in POI
5.	Kulaksizoglu (2013) (29)	Turkey (Konya)	43	33	36.83 ± 2.72	37.15 ± 1.88	NA	30.33 ± 5.59	29.62 ± 3.48	No	No	TC,TG,HDL,LDL	Serum TC,HDL and LDL increased, serum TG remained unchanged in POI
6.	Ates (2014) (22)	Turkey (Istanbul)	56	59	35.23 ± 4.58	35.47 ± 4.49	NA	25.79 ± 4.10	26.09 ± 3.80	No	No	TC,TG,HDL,LDL	Serum TC and HDL increased, serum TG and LDL remained unchanged in POI
7.	Ağaçayak (2016) (21)	Turkey (Diyarbakır)	30	30	28.9 ± 6.8	29.2 ± 5.0	NA	24.1 ± 4.2	23.2 ± 3.3	No	No	TC	Serum TC remained unchanged in POI
8.	AbdulAzeez (2018) (20)	Nigeria (Kwara)	50	40	26.4 ± 5.2	26.4 ± 5.5	NA	NA	NA	NA	NA	TC,TG,LDL,HDL	Serum TC,HDL,TG and LDL increased in POI
9.	Podfigurna (2018) (32)	Poland (Poznan)	56	68	30.70 ± 6.90	27.30 ± 4.50	NA	23.54 ± 4.55	28.62 ± 5.30	No	No	TC,TG,LDL,HDL	Serum TC,HDL increased, serum TG,LDL remained unchanged in POI
10.	Bozkaya (2020) (23)	Turkey (Ankara)	34	35	30.94 ± 5.58	28.53 ± 5.09	NA	25.90 ± 4.22	24.47 ± 3.89	No	No	TC,TG,LDL,HDL	Serum TC,TG,LDL and HDL remained unchanged in POI
11.	Cekici (2021) (24)	Turkey (Gaziantep)	66	73	32.5 ± 4.7	31.0 ± 4.9	NA	23.6 ± 4.3	22.7 ± 5.3	No	No	TC,TG,LDL,HDL	Serum TC,LDL increased,serum TG,HDL remained unchanged in POI
12.	Huang (2021) (27)	China (Hangzhou)	303	303	34.78 ± 5.42	34.68 ± 4.80	34.08 ± 5.21	21.44 ± 2.75	21.25 ± 2.92	No	No	TC,TG,LDL,HDL	Serum TG increased, serum HDL decreased, serum TC,LDL remained unchanged in POI

BMI, body mass index; HRT, hormone replacement therapy; OC, oral contraceptive; TC, Total cholesterol; TG, Triglyceride; HDL, high density lipoprotein; LDL, low density lipoprotein; NA, not available.

### Meta-Analysis Results

**Figure 2:**
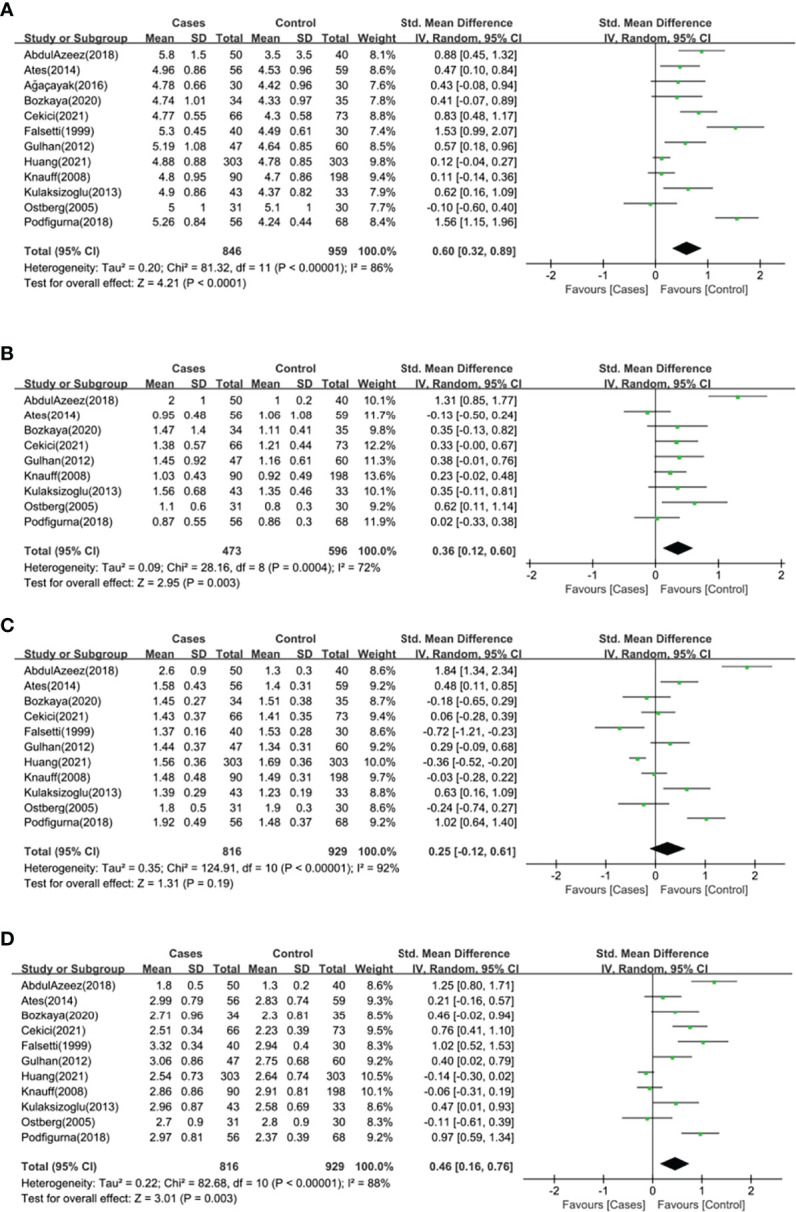
Forest plot of the levels of serum lipid profile in cases and healthy controls. Weights are from random effects analysis. **(A)** Meta-analysis of total cholesterol. **(B)** Meta-analysis of triglyceride. **(C)** Meta-analysis of high density lipoprotein. **(D)** Meta-analysis of low density lipoprotein. CI, confidence interval; SD, standard difference.

Twelve studies (*n*=1805 participants) compared the serum TC level between primary ovarian insufficiency women and healthy controls (**Figure 2A**), and there was significant heterogeneity among the studies (*I^2 ^= *86%; *P*<0.00001). Primary ovarian insufficiency was significantly associated with an increased serum TC level (SMD: 0.60; 95% CI: 0.32 to 0.89; *P*<0.0001).

Nine studies (*n*=1069 participants) compared the serum TG level between primary ovarian insufficiency women and healthy controls (**Figure 2B**), and there was significant heterogeneity among the studies (*I^2 ^= *72%; *P*=0.0004). Primary ovarian insufficiency was significantly associated with an increased serum TG level (SMD: 0.36; 95% CI: 0.12 to 0.60; *P=*0.003).

Eleven studies (*n*=1745 participants) compared the serum HDL level between primary ovarian insufficiency women and healthy controls (**Figure 2C**), and there was significant heterogeneity among the studies (*I^2 ^= *92%; *P*<0.00001). There was no significant association between primary ovarian insufficiency and serum HDL level (SMD: 0.25; 95% CI: -0.12 to 0.61; *P=*0.19).

Eleven studies (*n*=1745 participants) compared the serum LDL level between primary ovarian insufficiency women and healthy controls (**Figure 2D**), and there was significant heterogeneity among the studies (*I^2^* = 88%; *P*<0.00001). Primary ovarian insufficiency was significantly associated with an increased serum LDL level (SMD: 0.46; 95% CI: 0.16 to 0.76; *P=*0.003).

### Results of Subgroup Analysis

To search for the sources of heterogeneity and more accurately assess the differences between primary ovarian insufficiency women and healthy controls, subgroup analyses were conducted by geographical location, sample size, number of cases, mean BMI value of cases and mean age of cases.

As for geographical location (**Table 2**), a higher TC level was found in African (*p*<0.0001) and Turkish (*p*<0.00001) women with POI than in healthy controls, but not in Asian (*p*=0.16) and European cases (*p*=0.08), and the heterogeneity of Turkish subgroup decreased (*I^2 ^= *0%). A higher TG level was found in African (*p*<0.00001) and Turkish (*p*=0.02) women with POI than in healthy controls, but not in European cases (*p*=0.08), and the heterogeneity of Turkish (*I^2 ^= *22%) subgroup and European (*I^2 ^= *43%) subgroup decreased. A higher HDL level was found in African (*p*<0.00001) women with POI than in healthy controls, while a lower level was found in Asian (*p*<0.0001) cases but not in European(*p*=0.95) and Turkish (*p*=0.06) cases, and the heterogeneity of Turkish subgroup decreased (*I^2 ^= *53%). A higher LDL level was found in African (*p*<0.00001) and Turkish (*p*<0.00001) women with POI than in healthy controls, but not in Asian (*p*=0.09) and European (*p*=0.16) cases, and the heterogeneity of Turkish subgroup decreased (*I^2 ^= *16%).

**Table 2:** Subgroup analysis to investigate the relationship between geographical location and TC, TG, HDL, LDL.

Lipid parameters	Geographical location			
	Africa	Asia	Europe	Central Asia
**TC**				
**No. of studies**	1	1	4	6
***I^2^***	–	–	95%	0%
**SMD (95%CI)**	0.88 (0.45, 1.32)	0.12 (-0.04,0.27)	0.77 (-0.09,1.63)	0.58 (0.41,0.75)
***P***	*<*0.0001	0.16	0.08	*<*0.00001
**TG**				
**No. of studies**	1	0	3	5
***I^2^***	–	–	43%	22%
**SMD (95%CI)**	1.31 (0.85, 1.77)	–	0.24 (-0.03,0.51)	0.24 (0.04,0.44)
***P***	*<*0.00001	–	0.08	0.02
**HDL**				
**No. of studies**	1	1	4	5
***I^2^***	–	–	92%	53%
**SMD (95%CI)**	1.84 (1.34, 2.34)	-0.36 (-0.52,-0.20)	0.02 (-0.65,0.70)	0.26 (-0.01,0.52)
***P***	*<*0.00001	*<*0.0001	0.95	0.06
**LDL**				
**No. of studies**	1	1	4	5
***I^2^***	–	–	90%	16%
**SMD (95%CI)**	1.25 (0.80, 1.71)	-0.14 (-0.30,0.02)	0.45 (-0.17,1.07)	0.47 (0.27,0.66)
***P***	*<*0.00001	0.09	0.16	*<*0.00001

TC, Total cholesterol; TG, Triglyceride; HDL, high density lipoprotein; LDL, low density lipoprotein; SMD, standard mean difference.

As for sample size (**Table 3**), the TC level in the cases was higher than that in healthy controls (*p*<0.00001) with sample size<200, while in the subgroup with sample size≥200. There was no correlation of TC level in the cases and healthy controls (*p*=0.09), and this subgroup had no heterogeneity (*I^2 ^= *0%). The TG level in the cases was higher than that in healthy controls (*p*=0.008) with sample size<200, while it was not associated with POI (*p*=0.07) in the subgroup of sample size≥200. In the subgroup with sample size<200 (*p*=0.23) and sample size≥200 (*p*=0.21), there was no statistical difference in HDL level between the cases and healthy controls. LDL level was higher in women with POI than in healthy controls (*p*<0.00001) with sample size<200. However, in the subgroup with sample size ≥200, serum LDL was not associated with POI (*p*=0.10), and there was no heterogeneity (*I^2 ^= *0%) in this subgroup.

**Table 3:** Subgroup analysis to investigate the relationship between sample size, number of cases and TC, TG, HDL, LDL.

Lipid parameters	Sample size		Cases	
	<200	≥200	<50	≥50
**TC**				
**No. of studies**	10	2	6	6
***I^2^***	78%	0%	75%	92%
**SMD (95%CI)**	0.72 (0.43, 1.02)	0.11 (-0.02, 0.25)	0.57 (0.18, 0.96)	0.64 (0.22, 1.05)
***P***	*<*0.00001	0.09	0.004	0.003
**TG**				
**No. of studies**	9	1	4	5
***I^2^***	72%	–	0%	85%
**SMD (95%CI)**	0.35 (0.09, 0.61)	0.23 (-0.02, 0.48)	0.41 (0.19, 0.64)	0.33 (-0.06, 0.73)
***P***	0.008	0.07	0.0004	0.10
**HDL**				
**No. of studies**	10	2	6	5
***I^2^***	91%	79%	92%	93%
**SMD (95%CI)**	0.27 (-0.17, 0.70)	-0.21 (-0.53, 0.12)	0.27 (-0.41, 0.95)	0.43
***P***	0.23	0.21	0.21 (-0.24, 0.67)	0.35
**LDL**				
**No. of studies**	9	2	5	6
***I^2^***	72%	0%	59%	93%
**SMD (95%CI)**	0.61 (0.34, 0.87)	-0.11 (-0.25, 0.02)	0.45 (0.12, 0.77)	0.48 (0.03, 0.92)
***P***	<0.00001	0.10	0.007	0.04

TC, Total cholesterol; TG, Triglyceride; HDL, high density lipoprotein; LDL, low density lipoprotein; SMD, standard mean difference.

As for number of cases (**Table 3**), in the subgroup with cases <50 (*p*=0.004) and cases ≥50 (*p*=0.003), the TC level in women with POI was higher than that in healthy controls. Women with POI had a higher TG level than the healthy controls (*p*=0.0004) with cases <50, but not in the subgroup with cases≥50 (*p*=0.10),and there was no heterogeneity in this subgroup (*I^2^* = 0%).In the subgroup with cases <50 (*p*=0.43) and cases ≥50 (*p*=0.35), serum HDL was not associated with POI. In the subgroup with cases<50 (*p*=0.007) and cases≥50 (*p*=0.04), the LDL level in the cases was higher than in healthy controls, while the heterogeneity was reduced in the subgroup with the number of cases<50 (*I^2 ^= *59%).

As for the mean BMI value of cases (**Table 4**), patients with the mean BMI<25 kg/m^2^ had a higher TC level than the healthy controls (*p*=0.006). Meanwhile, the subgroup with the mean BMI≥25 kg/m^2^ had the same conclusion (*p*<0.00001), and no heterogeneity was found in this subgroup (*I^2 ^= *0%). The results showed that patients with the mean BMI<25 kg/m^2^ had a higher TG level than healthy controls (*p*=0.01), but not in the subgroup with the mean BMI≥25 kg/m^2^ (*p*=0.10), and the heterogeneity of this subgroup decreased (*I^2 ^= *36%). In the subgroup with the mean BMI of cases <25 kg/m^2^, there was no correlation of serum HDL in women with POI and healthy controls (*p*=0.42), and the same conclusion was found in the subgroup with the mean BMI of cases≥25 kg/m^2^ (*p*=0.05), however, the heterogeneity of this subgroup decreased (*I^2 ^= *55%).The results showed that patients with the mean BMI<25 kg/m^2^ had a higher LDL level than healthy controls (*p*=0.02), meanwhile, the subgroup with the mean BMI≥25 kg/m^2^ had the same conclusion (*p*=0.0006), and no heterogeneity was found in this subgroup (*I^2 ^= *0%).

**Table 4:** Subgroup analysis to investigate the relationship between mean BMI value of cases, mean age of cases and TC, TG, HDL, LDL.

Lipid parameters	BMI (kg/m^2^)		Age (years)	
	<25	≥25	<35	≥35
**TC**				
**No. of studies**	8	4	9	3
***I^2^***	92%	0%	90%	0%
**SMD (95%CI)**	0.62 (0.18, 1.06)	0.52 (0.31, 0.73)	0.63 (0.26, 0.99)	0.54 (0.31, 0.78)
***P***	0.006	*<*0.00001	0.0008	*<*0.00001
**TG**				
**No. of studies**	5	4	6	3
***I^2^***	82%	36%	77%	53%
**SMD (95%CI)**	0.48 (0.09, 0.86)	0.22 (-0.04, 0.48)	0.45 (0.13, 0.78)	0.19 (-0.15, 0.52)
***P***	0.01	0.10	0.006	0.28
**HDL**				
**No. of studies**	6	4	8	3
***I^2^***	94%	55%	94%	0%
**SMD (95%CI)**	0.21 (-0.30, 0.73)	0.32 (0.00, 0.63)	0.17 (-0.30, 0.63)	0.45 (0.22,0.68)
***P***	0.42	0.05	0.48	0.0001
**LDL**				
**No. of studies**	7	4	8	3
***I^2^***	92%	0%	91%	0%
**SMD (95%CI)**	0.51 (0.07, 0.95)	0.36 (0.19, 0.57)	0.50 (0.10, 0.90)	0.34 (0.11, 0.57)
***P***	0.02	0.0006	0.01	0.003

BMI, body mass index; TC, Total cholesterol; TG, Triglyceride; HDL, high density lipoprotein; LDL, low density lipoprotein; SMD ,standard mean difference.

As for the mean age of cases (**Table 4**), women under the age of 35 with POI had a higher TC level than healthy controls (*p*=0.0008), and the same conclusion was found in women over 35 with POI (*p*<0.00001), meanwhile, there was no heterogeneity in this subgroup (*I^2 ^= *0%). The results showed that women under the age of 35 with POI had a higher TG level than healthy controls (*p*=0.006), but in the subgroup with the mean age of cases ≥35, TG level was not associated with POI (*p*=0.28), and the heterogeneity was reduced in this subgroup (*I^2 ^= *53%). In the subgroup with the mean age of cases<35, serum HDL was not associated with primary ovarian insufficiency (*p*=0.48). While in the subgroup with the mean age of cases ≥35, the HDL level in the cases was higher than that in healthy controls (*p*=0.0001), and there was no heterogeneity in this subgroup (*I^2 ^= *0%). In the subgroup with the mean age of cases <35 (*p*=0.01) and ≥35 (*p*=0.003), the LDL level in the cases was higher than that in healthy controls, and there was no heterogeneity in the subgroup with the mean age ≥35 (*I^2 ^= *0%).

### Sensitivity Analysis

As for TC, HDL and LDL levels, there was no qualitative change in the total effect size after removing the studies one by one, indicating that the meta-analysis results were stable and reliable.As for TG level, the results of this meta-analysis were weakly stable and sensitive to AbdulAzeez’s study (20). After removing this study, the heterogeneity decreased (*I^2 ^= *19%; *p*=0.28), TG level in women with POI was still higher than that in healthy controls (SMD: 0.24; 95% CI: 0.09 to 0.38; *P*=0.001).

## Publication Bias

**Figure 3:**
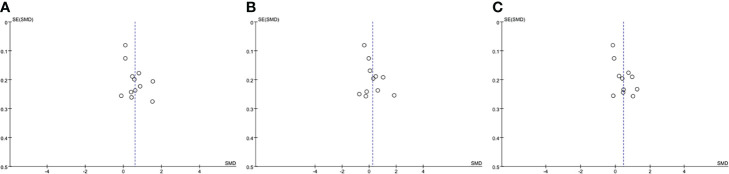
Funnel plot analysis to detect publication bias. **(A)** Funnel plot for total cholesterol. **(B)** Funnel plot for high density lipoprotein. **(C)** Funnel plot for low density lipoprotein. SMD, standard mean difference.

The funnel plot method was used to detect publication bias, regarding TC, HDL and LDL, the shape of the funnel plots was not obviously asymmetric (**Figure 3**), indicated that there was no significant evidence of publication bias in this meta-analysis. The publication bias for TG was not assessed, as the Cochrane Handbook for Systematic Reviews of Interventions (www.cochranehandbook.org) stated that the test for publication bias yields unreliable results when <10 studies were included.

## Discussion

To our knowledge, this is the first meta-analysis to explore the relationship between lipid profile and POI. A total of 12 studies were selected in this meta-analysis, and 1805 women were represented. The subgroup analyses based on region, sample size, number of cases, mean BMI value of cases and mean age of cases were performed. This meta-analysis indicated that levels of serum lipid profile were remarkably altered in patients with primary ovarian insufficiency: levels of serum TC, TG and LDL in women with POI were significantly higher than that in healthy controls, however, no significant difference was observed regarding the level of serum HDL between women with POI and healthy controls.

Women with POI present several risk factors for the occurrence and development of CVD: autonomic and endothelial dysfunction, insulin dysfunction, abnormal lipid profile (33), genetic variants associated with early menopause (34, 35). Diminished ovarian response to FSH is thought to cause the occurrence and development of atherosclerosis based on non-human primate models (36) and studies of hypothalamic angiography (37). Moreover, women with POI have been demonstrated to have impaired endothelial function regarded as a precursor to atherosclerosis (31, 38). It has been reported that women during the menopause transition exhibit abnormal lipid profile, including increased serum levels of TC, LDL, TG and decreased level of HDL (4, 39). In the same way, early impairment of ovarian function result in earlier or worse lipid profile alterations which lead to CVD in women with POI. Futhermore, genome-wide association studies have identified genetic overlap between the age associated decline in reproduction, lipid regulation and glucose regulation (40, 41). Hence, early menopause is associated with the subsequent CVD, conversely, women who experience CVD events prematurely (before age 35 years) have POI due to the genetic factors and the compromised vasculature following CVD events (42).

The beneficial effects of intact ovarian function on lipid profile are generally ascribed to estradiol. Endogenous estradiol acts like an antioxidant, which has vasodilating and protective effects on cardiovascular system. It has also proven that estradiol promotes the degradation and excretion of blood fat, decreases the levels of serum TC and LDL, alters the distribution of TC in the body and reduces the accumulation of TC in the artery walls (43). Meanwhile, estradiol inhibits the activity of liver lipase, decelerates the degradation rate of HDL, accelerates the removal of LDL particles by liver and reduces the levels of serum TC and LDL by strengthening the activity of LDL receptors, thus improving lipid metabolism (44). In addition, estradiol deficiency leads to the activation of the renin-angiotensin system, the upregulation of the vasoconstrictor endothelin and the impairment of nitricoxide-mediated vasodilation. Endothelin and angiotensin II augment the oxidative stress, which may further contribute to atherosclerosis (45). Meanwhile, decreased estradiol reduces the release of nitric oxide by vascular endothelial cells by reducing the sensitivity of estrogen receptors, thereby increases the cardiac load (46). Thus, women with POI have a higher rate of CVD morbidity and mortality later in life due to estradiol deficiency for longer than women with natural menopause (38, 47, 48).

Hormone replacement therapy (HRT) has been recommended by international guidelines for women with POI for many years (12). HRT is an exogenous supplement, which can relieve clinical symptoms, prevent the occurrence and development of diseases, and reduce the long-term complications with high disability rate and fatality rate (e.g., cardiovascular disease), and improve living standards (12) of women with POI. It has been reported that HRT can regulate lipid metabolism, strengthen LDL clearance, reduce the levels of serum TC, TG, LDL, improve lipoprotein composition ratio, and reduce the risk of cardiovascular events (49). HRT and simvastatin have similar lipid-regulating effects (50). In addition, the second report of the National Cholesterol Education Program recommends HRT as a treatment for dyslipidemia in postmenopausal women. Importantly, a 1-mg/dl increase in HDL level may be associated with a 3%~5% decrease in CVD, and again a 1% reduction in TC level equals a 2% reduction in CVD, however, this could only be achieved with HRT (51). Unfortunately, the acceptance rate of HRT in women with POI is extremely low (52).

Subgroup analysis was conducted to further explore the source of heterogeneity and more accurately evaluate the correlation between lipid profile and POI. Heterogeneity in this meta-analysis of the correlation between TC and POI might come from region, sample size, mean BMI value of cases and mean age of cases. The correlation between TG and POI might come from region, sample size, number of cases, mean BMI value of cases and mean age of cases. The correlation between HDL and POI might come from region, mean BMI value of cases and mean age of cases. And the correlation between LDL and POI might come from region,sample size, number of cases, mean BMI value of cases and mean age of cases. (1) Because there is considerable overlap in Turkey of people with European ethnicity as well as west Asian (53), the regions in this meta-analysis were divided into Europe, Africa, Asia and Central Asia. People in different regions have different dietary habits, lifestyles and economic levels, which have different effects on levels of serum lipid profile; (2) all references included in this meta-analysis were designed as case-control studies. In case-control study, if the sample size or the number of cases is enormous, the research results will be affected by more confounders; (3) BMI≥25 kg/m^2^ is diagnosed as overweight (54), overweight has significant impact on serum lipid profile, therefore, BMI value may be one of the sources of heterogeneity; and (4) ovarian reserve function is related to physiological age, in the reproductive domain, age>35 is considered as advanced age, and ovarian function significantly declines (55), thus physiological age is regarded as one of the sources of heterogeneity.

Part of the sources of heterogeneity was found by subgroup analysis, however, the heterogeneity was still obvious, hence, sensitivity analysis was conducted. In this meta-analysis,the combined results of the correlation between TG level and POI were sensitive to AbdulAzeez’s study (20), and the *P* values of the combined results were all less than 0.05 before and after the exclusion of the references. However, after excluding the references, the heterogeneity decreased (*I^2^* decreased from 72% to 19%), therefore, AbdulAzeez’s study (20) had significant impact on the heterogeneity of the combined results. After careful reading of the references, we found TG level in the study was estimated using glycerol-3 phosphate oxidase method, distinguishing from other studies using enzymatic and colorimetric methods. In addition, BMI value of the participants included in the study was not mentioned, and these confounders might cause heterogeneity. Sensitivity analysis revealed that the combined results of the correlation between TC, HDL, LDL levels and POI were stable and reliable. Moreover, no significant evidence of publication bias was found in this study, which also increased the credibility of this meta-analysis.

Nevertheless, a number of limitations in this meta-analysis should be acknowledged. Firstly, obvious heterogeneity existed among the original studies due to differences in sample size, background of subjects, diagnostic criteria of POI, and methods used to detect the levels of serum lipid profile. Secondly, the subgroup analyses of HRT using and mean age at diagnosis of POI were unable to be conducted due to the incomplete data. While the heterogeneity may relate to duration of POI rather than age per se, and the HDL level may be similar between POI and healthy controls due to the effect of HRT. Thirdly, the main confounders (e.g., diet or physical activity) influencing the lipid profile of patients in original studies were not adjusted. Finally, the references included in this study were all case-control studies, which would limit causal inference. Therefore, more cohort studies are needed to predict how the lipid profile in women with POI will develop over time. For these reasons, we recommend that our conclusions should be viewed conservatively.

In summary, this systematic review and meta-analysis demonstrated that the levels of serum TC, TG and LDL were significantly higher in subjects with POI. These mean that women who loss of ovarian function at a very young age will lead to some degree of changes in the lipid profile, increasing the risk of CVD. Therefore, we recommend that lipid profile should be evaluated and comprehensively studied to ensure that early medical intervention (especially HRT) and minimize the risk of CVD morbidity and mortality associated with dyslipidemia in patients with POI. Moreover, further studies are required to verify the relationship between serum HDL level and POI.

## Data Availability Statement

The raw data supporting the conclusions of this article will be made available by the authors, without undue reservation.

## Author Contributions

LH, HW, MS, WK and MJ: literature search, screening, and data extraction. LH, HW, MS, WK: data analysis and results visualization. LH and HW: manuscript draft. MJ: manuscript modification. All authors reviewed the final version of the manuscript and approve it for publication. All authors contributed to the article and approved the submitted version.

## Funding

This work was supported by grant from the National Natural Science Foundation of China (No. 81904240) and New Teacher Foundation of Beijing University of Chinese Medicine (No. 2021-JYB-XJSJJ-011).

## Conflict of Interest

The authors declare that the research was conducted in the absence of any commercial or financial relationships that could be construed as a potential conflict of interest.

## Publisher’s Note

All claims expressed in this article are solely those of the authors and do not necessarily represent those of their affiliated organizations, or those of the publisher, the editors and the reviewers. Any product that may be evaluated in this article, or claim that may be made by its manufacturer, is not guaranteed or endorsed by the publisher.
